# An Extremely Effective Spatial Pyramid and Pixel Shuffle Upsampling Decoder for Multiscale Monocular Depth Estimation

**DOI:** 10.1155/2022/4668001

**Published:** 2022-08-01

**Authors:** Huilan Luo, Yuan Chen, Yifeng Zhou

**Affiliations:** School of Information Engineering, Jiangxi University of Science and Technology, Ganzhou 341000, China

## Abstract

To estimate the accurate depth from a single image, we proposed a novel and effective depth estimation architecture to solve the problem of missing and blurred contours of small objects in the depth map. The architecture consists of Extremely Effective Spatial Pyramid modules (EESP) and Pixel Shuffle upsampling Decoders (PSD). The results of this study show that multilevel information and the upsampling method in the decoders are essential for recovering the accurate depth map. Through the model we proposed, competitive performance compared with state-of-the-art methods in terms of reconstruction of object boundaries and the detection rate of small objects has been demonstrated. Our approach has wide applications in higher-level visual tasks, including 3D reconstruction and autonomous driving.

## 1. Introduction

Monocular depth estimation is a long-standing task, which aims to predict the continuous depth value of each pixel from a single RGB image. This task has a wide range of application in various fields, such as scene understanding [1], scene segmentation [2], 3D reconstruction [3], and simultaneous localization and mapping (SLAM) [4]. Traditional depth estimation methods of image-based depth estimation are usually based on a binocular camera, which calculates the disparity of two 2D images (taken by a binocular camera) through stereo matching and triangulation to obtain a depth map. However, the binocular depth estimation method requires at least two fixed cameras, and it is difficult to capture enough features in the image to match when the scene has less or no texture. Therefore, researchers turn their attention to monocular depth estimation. Monocular depth estimation uses only one camera to obtain an image, which does not require additional complicated equipment and professional techniques. Thus, there has been an increasing demand for monocular depth estimation in recent years.

With the great success of deep learning, researchers have studied a number of various monocular depth estimation methods based on convolutional neural networks (CNN). Eigen et al. [5] first proposed a multiscale deep network to regress dense depth maps from coarse to fine. Laina et al. [6] proposed a fully convolutional residual network, which explores a new upsampling method to obtain more accurate depth predictions. Liu et al. [7] proposed a deep convolution neural field, which combines the depth convolution neural network with the continuous conditional random field to extract the structural information of features. In recent years, there have been new advances in monocular depth estimation. Wofk et al. [8] proposed an efficient and lightweight encoder-decoder network architecture and applied network pruning to further reduce computational complexity and latency for real-time monocular depth estimation. In literature [9], an attention mechanism and a multiscale feature fusion dense pyramid were used to further improve estimated depth maps of distant small-scale objects. In literature [10], an adversarial loss was introduced into the training stage of self-supervised depth estimation to optimize the depth network with high-level information.

Although monocular depth estimation research has made great progress, there are still some problems such as missing small objects and distortions in object shapes. Some works address the above issues by introducing auxiliary information such as geometric constraints and semantic information [11]. Others use feature fusion methods to improve depth estimation of small objects [9]. The CNN-based methods use a convolutional neural network to extract image features and then regress the features into image depth values, so the quality of extracted features directly affects the effect of monocular depth estimation. Inspired by this perception, we propose a novel U-shape network based on EESP skip connection modules and an upsampling method based on PSD modules. These modules can result in fewer parameters, clearer object contours, less object distortion, and fewer small objects missing.The main contributions can be summarized as follows:

- We design the Extremely Effective Spatial Pyramid (EESP) connection module as the skip connection of the U-shaped network. The EESP connection module transfers the multiscale features of different layers of the backbone network into the PSD module to supplement multilevel comprehensive information so as to obtain a better depth estimation effect. It is extremely effective in terms of performance improvement.
- We introduce the Pixel Shuffle upsampling Decoders (PSD) module for the decoder to improve the resolution of feature maps and fully fuse feature information of different receptive fields. Compared with other upsampling methods, our method does not introduce extra parameters and can better alleviate the edge blur and artifacts caused by information loss.

## 2. Related Works

### 2.1. U-Shaped Networks

Since the FCRN [6] network was proposed, the encoder-decoder structure has been adopted in most monocular depth estimation methods. The encoder downsamples the input image several times to extract the global features, and the decoder upsamples the feature map to get the depth map. However, only relying on the final high-level features to estimate the depth of each pixel, the performance is not desirable because it lacks local detail information. Therefore, more recently, the U-shaped network, for example, U-Net, is commonly used, in which the decoder utilizes features from all layers by adding shortcut connections between the corresponding decoder layers and the encoder layers. In work [8], the multilevel features extracted from the encoder are directly added to the decoder. Their experimental results demonstrate that the enhancement of local features improves the accuracy of local depth in the prediction maps. Different from them, in this paper, the spatial pyramid EESP module is added to the skip connections of the U-Net network structure to fuse different scale-level features from different encoder layers.

### 2.2. Upsampling Methods

Depth estimation models usually use a backbone network such as ResNet [12] to extract the features of the input image, and the resolution of the output features is usually small, which will limit the resolution of the depth prediction map. Therefore, it is necessary to use encoder-decoder structure with upsampling operations at the decoder part to improve the resolution of the features. Commonly used upsampling methods are interpolation, deconvolution, and pixel shuffle.

Bilinear interpolation is the most commonly used interpolation method [13, 14], but it will dilute the feature information, thus blurring the image edges, and ultimately affect the effect of depth estimation. Deconvolution is also one of the popular upsampling methods. Its advantage is that it can improve the upsampling effect through training, but multiple deconvolutions may produce artifacts [15]. Therefore, some works [6, 8] add convolution layers after deconvolution to alleviate the artifacts caused by continuous deconvolution to a certain extent.

Pixel shuffle, also known as subpixel convolution, is widely used in the field of image super-resolution processing [16, 17]. This method improves the resolution of the feature maps by reducing the number of channels of features, which also results in fewer parameters for subsequent convolution operations. Pixel shuffle retains all the feature information, which can better alleviate the edge blur and artifacts caused by information loss. Due to the advantages of pixel shuffle, some researchers have applied it to other computer vision areas, such as image reconstruction [18] and semantic segmentation [19]. In order to improve the resolution of output feature maps and further learn feature fusion, this paper uses pixel shuffle operation in the decoder. By rearranging the input features, pixel shuffle realizes the feature fusion between different channels, so it can also play the role of feature fusion.

### 2.3. Depthwise Separable Convolution

The depthwise separable convolution [20] consists of two parts: a depthwise convolution and a pointwise convolution. The depthwise convolution is to convolute each channel independently, while the pointwise convolution is to fuse features across channels. The depthwise separable convolution is widely used in lightweight networks such as MobileNet [21] and FastDepth [8] due to its fewer parameters. Moreover, compared with the standard convolution, the depthwise separable convolution pays more attention to the fusion of features in a single channel. Since the pixel shuffle operation improves the resolution by reshaping multiple channels into one channel, this paper uses the depthwise separable convolution on the output of pixel shuffle layer not only to reduce the number of network parameters but also to improve the feature fusion effect of pixel shuffle. In addition, the dilated convolution [22] can increase the receptive field of the convolution kernel without introducing extra parameters. Therefore, in this paper, depthwise dilated separable convolutions are used at the decoder part to obtain a larger receptive field and multiscale fusion feature while keeping as few parameters as possible.

## 3. Method

### 3.1. The Overview of Our Method

**Figure 1:**
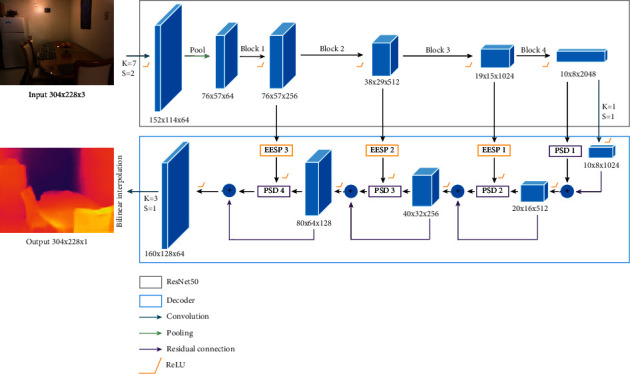
The framework of our proposed PSDNet.

In recent years, the U-shaped network [8] has been commonly used in most monocular depth estimation methods. On this basis, we propose the pixel shuffle encoder-decoder convolution neural network (PSDNet). As shown in Figure 1, PSDNet uses ResNet [12] as the backbone network to extract features from the input image. Three EESP connection modules are used to transfer information from the three residual blocks of the feature extraction backbone to the upsampling modules of the decoder network. The decoder network contains four upsampling modules, that is, pixel shuffle decoder (PSD). Besides, the residual connections between every two adjacent PSD modules, which consist of bilinear interpolation and a 5 × 5 convolution layer, are designed to enhance information sharing and alleviate the gradient vanishing problem. Finally, the depth map is obtained by using a 3 × 3 convolution layer on the output of the last PSD module, and then the resolution of the prediction map is increased to the size of the input image using bilinear interpolation.

### 3.2. EESP Connection Module

**Figure 2:**
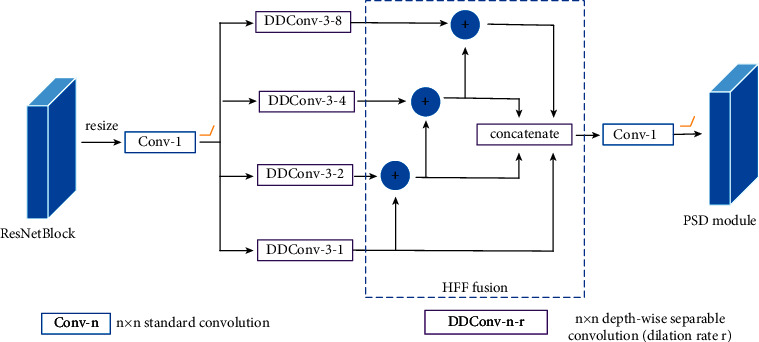
The architecture of the EESP3 connection module.

Motived by the feature fusion method of the spatial pyramid in lightweight network [23], we design different EESP skip connection modules to bring more comprehensive information to the decoder. The dilated convolution [22] increases the receptive field while avoiding a surge in the number of parameters. The depthwise separable convolution [20, 21] can also reduce the number of parameters by separating it into a depthwise convolution and a pointwise convolution. The combination of the two methods not only increases the receptive fields but also reduces the number of parameters. Therefore, it is much lighter and more efficient than other feature fusion methods. The EESP module extracts features using depthwise separable convolutions with different dilation rates and fuses the extracted features using the hierarchical feature fusion method HFF [23]. In HFF, feature maps from the branch with the lowest dilation rate are combined with the feature maps from the branch with the next highest dilation rate, and then all the features are concatenated and input into a 1 × 1 convolution layer to further fuse the features (see Figure 2). HFF enhances the convolution of small dilation rates and thus can effectively alleviate the grid artifacts [15] caused by dilated convolutions. In this paper, the EESP module is added to the skip connection of the U-Net structure to supply the lower-level features for decoder. Considering that the resolution of different residual blocks in the backbone network is different, different EESP modules are designed to make full use of these features and improve the performance of depth estimation.

As shown in Figure 1, there are four residual blocks at the encoder part. Except for the last residual block, the output features of the other three residual blocks are input into three EESP skip connection modules, respectively (denoted as EESP3, EESP2, and EESP1), to connect to the decoder. Features extracted from shallower residual blocks have larger feature resolution and fewer channels, and so it is considered that they contain more local information reflecting the depth of details. On the other hand, the deeper features have smaller resolution and so more global depth information. In order to balance the local and global information, the dilation rates of EESP connection modules connecting different residual blocks are set to different values. The dilation rates of depthwise dilated separable convolutions in the EESP3 connection module are set to 1, 2, 4, and 8. The dilation rates of the EESP2 connection module are set to 1, 2, and 4, while the dilation rates in the EESP1 connection module are set to 1 and 2.

The EESP connection module not only achieves the unification of resolutions and channel number of multiscale features but also can further learn extra features with only a few parameters. The main process in Figure 2 is shown as follows:

(1) $$\begin{array}{c} X_{j}={\text{DDConv}}_{3\times 3,i}\left(X\right),\;j=1,\;2,\;3,\;4,\\ \text{EESP}3=\Phi_{\text{HFF}}\left(X_{1},X_{2},X_{3},X_{4}\right), \end{array}$$

where *X* denotes the input feature maps from the corresponding residual block of the backbone network, DDConv_3×3,*i*_ denotes 3 × 3 depthwise dilated separable convolutions with dilation rates *i*, and *i* is set to 1, 2, 4, and 8, respectively. Φ_HFF_ denotes fuse features of different branches. Feature maps from the branch with the lowest dilation rate are combined with the feature maps from the branch with the next highest dilation rate. Then, the features of all branches are concatenated as the output of HFF.

### 3.3. Pixel Shuffle Decoder

Pixel shuffling is widely used in the field of image super-resolution processing [17]. By rearranging the input features, pixel shuffling can not only play the role of upsampling but also reduce the number of channels. The reduction of the number of channels can also greatly reduce the parameters of the subsequent convolution layer. In addition, compared with other upsampling methods (such as deconvolution [15]), it has no parameters. Therefore, we design the PSD module based on pixel shuffling.

**Figure 3:**
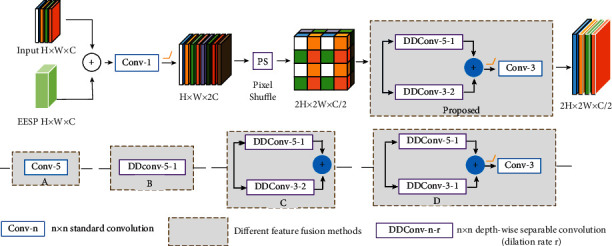
The overview of the PSD module.

The structure of the PSD modules (denoted as PSD2, PSD3, and PSD4 in Figure 1) is shown in Figure 3. The PSD module first adds the features from the previous PSD module and the output features of the corresponding EESP connection module. For the PSD1 (see Figure 1), there is only one input, which is the deepest level features output by the last residual block, and the other structures are the same as in Figure 3. The added features double the number of channels using a 1 × 1 convolution layer, and then the pixel shuffle unit changes the feature map to 2*H* × 2*W* × *C*/2; that is, the length and width of the input features are doubled, while the number of channels is reduced to one-fourth of the original. Pixel shuffle can improve the resolution and reduce the number of channels. At the same time, it can achieve the effect of feature fusion by disrupting the feature values. However, pixel shuffling also destroys the connections between feature values in each channel, so this paper uses depthwise separable convolution to reconstruct the connections between feature values. In the proposed PSD module, after the pixel shuffle layer, a 5 × 5 depthwise separable convolution and a 3 × 3 depthwise separable convolution with a dilation rate of 2 are designed in parallel to establish new connections in each channel. The feature maps of these two convolution branches are summed and then further fused using a 3 × 3 convolution to get the final output of the PSD module. The process is shown as follows:

(2) $$\begin{array}{c} Z=\varphi_{ps}\left({\text{Conv}}_{1\times 1}\left(Y+x\right)\right),\\ \text{PSD}={\text{Conv}}_{3\times 3}\left({\text{DDConv}}_{5\times 5,1}\left(Z\right)+{\text{DDConv}}_{3\times 3,2}\left(Z\right)\right), \end{array}$$

where *x* denotes the input feature maps from the previous PSD module and its residual connection, *Y* denotes the input feature from the EESP module, and *φ*_*ps*_ denotes pixel shuffle operation.

### 3.4. Loss Function

In order to improve the sharpness of the object edges in the predicted depth map, the loss function *L*_total_ is composed of three parts: the *L*_1_ loss function, the gradient loss function, and the *L*_2_ regularization term, as shown in equation (5). *α* is set to 0.5 and *β* to 0.0001 in our experiments.

(3) $$\begin{array}{c} L_{\text{total}}=L_{1}+\alpha L_{\text{gradient}}+\beta L_{2-\text{normal}}, \end{array}$$

where *L*_1_ loss function calculates the absolute error between the predicted value ${\hat{y}}_{i}$ and the true value *y*_*i*_, as shown in equation (2), where *i* is the pixel index and *N* denotes the number of pixels in the depth map. *L*_1_ loss function measures the overall error between the prediction and the ground truth, so minimizing the *L*_1_ loss function makes the predicted depth map accurate.

(4) $$\begin{array}{c} L_{1}=\frac{1}{N}\sum_{i=1}^{N}\left|y_{i}-\hat{y_{i}}\right|. \end{array}$$

The gradient loss function is shown in equation (5), where ${▽}_{h}{\hat{y}}_{i}$ and ${▽}_{v}{\hat{y}}_{i}$ denote the horizontal gradient and vertical gradient of the predicted depth map, respectively. ▽_*h*_*y*_*i*_ and ▽_*v*_*y*_*i*_ denote the horizontal gradient and vertical gradient of the true depth map, respectively. The gradient of the depth map reflects the change rate of depth values. This loss function is designed to make the depth change of the prediction map more real.

(5) $$\begin{array}{c} L_{\text{gradient}}=\frac{1}{2N}\sum_{i=1}^{2N}\left(\left|{▽}_{h}y_{i}-{▽}_{h}\hat{y_{i}}\right|+\left|{▽}_{v}y_{i}-{▽}_{v}\hat{y_{i}}\right|\right). \end{array}$$

## 4. Experimental Results and Analysis

### 4.1. Experimental Settings

#### 4.1.1. Dataset

We evaluate our method on the NYU depth V2 [24] indoor scene dataset, which is commonly used for monocular depth estimation. The NYU depth V2 contains 464 indoor scenes with 249 scenes for training and 215 scenes for testing.

#### 4.1.2. Implementation Details

To improve the accuracy of our proposed network and prevent overfitting, an online data augmentation method is adopted. The specific operations of data augmentation are randomly rotating the RGB-D image with a rotation angle range of [−5, 5]; randomly adjusting the brightness, contrast, and saturation of the RGB image in the range of [0.6, 1.4]; and randomly flipping the RGB image and the depth truth map horizontally with 50% probability. We train on a single NVIDIA GeForce RTX 2080Ti with 11 GB of GPU memory. The experiments are conducted on the TensorFlow framework. The Adam optimizer is used for training, with an initial learning rate of 0.0001 and a decaying learning rate of 0.1 per 5 iterations. The batch size is set to 8, and the maximum number of iterations is 30.

#### 4.1.3. Evaluation Metrics

The following evaluation metrics are used to measure the performance of monocular depth estimation methods: absolute relative error (rel, lower is better), root mean squared error (rms, lower is better), log mean error (log  10, lower is better), and threshold accuracy (*δ*_1_, *δ*_2_, and *δ*_3_, higher is better). The functional expressions of the evaluation metrics are shown as follows.

(6) $$\begin{array}{c} \text{rel}=\frac{1}{N}\sum_{i=1}^{N}\left|\frac{y_{i}-\hat{y_{i}}}{y_{i}}\right|,\\ \text{rms}=\sqrt{\frac{1}{N}\sum_{i=1}^{N}{\left|y_{i}-\hat{y_{i}}\right|}^{2}},\\ \log\;10=\frac{1}{N}\sum_{i=1}^{N}\left|\mathrm{lg}y_{i}-\mathrm{lg}\hat{y_{i}}\right|,\\ \delta =\max\left(\frac{y_{i}}{\hat{y_{i}}},\frac{\hat{y_{i}}}{y_{i}}\right)<T,\;T\in \left(1.25,1.25^{2},1.25^{3}\right). \end{array}$$

#### 4.1.4. Compared with Other Advanced Methods

The depth estimation test results on the NYU depth V2 dataset of our method and other state-of-the-art monocular depth estimation methods based on deep learning in recent years are reported in Table 1. Other methods involved in the comparisons are the full convolutional network method of Laina et al. [6], the conditional random field optimization of superpixel depth proposed by Liu et al. [7], the cascaded conditional random field depth optimization method of Xu et al. [25], the efficient and lightweight encoder-decoder network architecture proposed by Wofk et al. [8], the gradient optimization proposed by Li et al. [26], the multiscale feature fusion method of Xu et al. [27], the augment ordinal depth relationship methods of Cao et al. [28], the method based on the geometric cues and scene parsing of He et al. [11], the successive encoder-decoder style subnetworks proposed by Dong et al. [29], and the attention mechanism and multiscale feature fusion method proposed by Xu et al. [9]. Different from the feature fusion methods [9], our multiscale feature fusion method transfers the features of different receptive fields to the decoder network, constructing the connections between the encoder and the decoder. Among them, the methods of Laina et al. [6], Wofk et al. [8], Xu et al. [9], Cao et al. [28], Dong et al. [29], and this paper do not perform other additional refinement steps, while Liu et al. [7], Xu et al. [25], and Xu et al. [27] used conditional random fields to do postprocessing. Li et al. [26] used image gradients for depth map optimization, and He et al. [11] used the geometric constraints and semantic information of the scene to alleviate the ambiguity in monocular depth estimation. As can be seen from the results in Table 1, the method in this paper achieves competitive results in all indicators.

**Figure 4:**
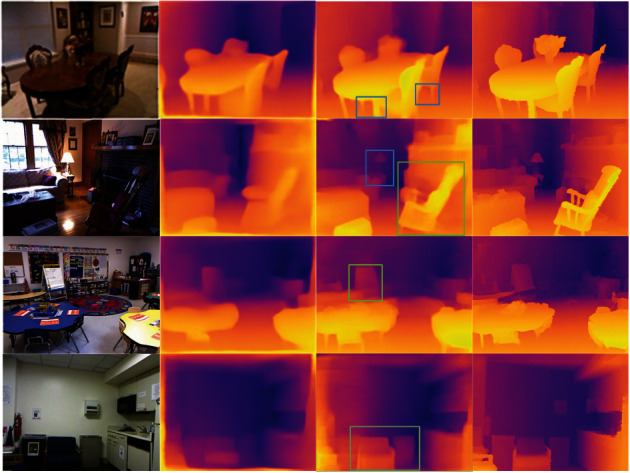
Qualitative results on the NYU depth V2 dataset. (a) RGB inputs; (b) results of Laina et al. [6]; (c) results of our method; (d) ground truth depth maps. The brighter the color of the pixel points, the smaller the depth value, and the darker the color, the larger the depth value.

Figure 4 illustrates some depth estimation results of our method and Laina et al. [6], where column (a) indicates the input RGB image, column (b) is the predicted depth map of Laina et al. [6], column (c) is the depth prediction map of this paper, and column (d) indicates the depth truth map. The brighter the color of the pixel points, the smaller the depth value, and the darker color, the larger the depth value. Observing the prediction maps in Figure 4, we can find that the performance of our method is better than that of Laina et al. [6] in terms of local depth values. For example, the depth estimation results marked by the blue rectangular box in column (c) indicate that our method can better predict the depth of small objects such as chair legs and table lamps. And the results within the green box in column (c) shows that the prediction map of our method has clearer edges than that of Laina et al. [6]. Specifically, the outline of the rocking chair in the second row can be clearly seen in our predicted depth map.

## 5. Ablation Experiments

In this section, we conduct experiments to illustrate the effectiveness of each component of our PSDNet.

### 5.1. Comparison of Different EESP Modules

In this experiment, we compare the depth estimation effects of several other structure options of the three EESP modules (see Figure 1), and the experimental results are shown in Table 2. In Table 2, “3-EESP” indicates that the three EESP connection modules adopt the same design as in Figure 2; that is, they all use four depthwise separable convolutions with dilation rates of 1, 2, 4, and 8, respectively. “EESP-4” indicates one depthwise separable convolution with the dilation rate of 1 for EESP1, two depthwise separable convolutions with dilation rates of 1 and 2, respectively, for EESP2, and three depthwise separable convolutions with dilation rates of 1, 2, and 4, respectively, for EESP3. “EESP-HFF” indicates that the HFF fusion is removed from the EESP modules, and others remain the same as in our proposed design, in which EESP1 has two depthwise separable convolutions with dilation rates of 1 and 2, respectively, EESP2 has three depthwise separable convolutions with dilation rates of 1, 2, and 4, respectively, and EESP3 has four depthwise separable convolutions with dilation rates of 1, 2, 4, and 8, respectively.

From the comparison results with “3-EESP” and “EESP-4,” it can be seen that our proposed design schema obtains the best performance due to features of different receptive fields fused in a complementary fashion. Compared with the experimental results of “EESP-HFF,” the rms is improved after using HFF fusion, while the rel remains unchanged. Since the rel is more sensitive to the smaller depth area than the rms, the improvement of rms can prove that HHF fusion can slightly improve the prediction accuracy of the larger depth area.

### 5.2. Comparison of Different Feature Fusion Methods in PSD Modules

In order to study how different fusion methods after pixel shuffling in PSD modules affect the performance of the model, we compare different design options. The results are compared in Table 3, where “A” indicates that only the standard 5 × 5 convolution is used for feature fusion after pixel shuffling; “B” indicates that only the depthwise separable convolution with a kernel size of 5 × 5 and a dilation rate of 1 is used for feature fusion after pixel shuffling; “C” means that, after pixel shuffling, the features are input in parallel into a depthwise separable convolution of 3 × 3 with a dilation rate of 2 and a depthwise separable convolution of 5 × 5 with a dilation rate of 1, and then simply add the outputs of these two branches as the result features; “D” is similar to the method proposed in Figure 3, except that the dilation rate of the 3 × 3 depthwise dilated separable convolution is reduced to 1. From the results in Table 3, it can be found that the “proposed” structure in Figure 3 has the best performance. It demonstrates that the proposed method can further integrate the features of different receptive fields and reconstruct the relationship between different channels.

### 5.3. Ablation Experiments on Decoder Network

**Figure 5:**
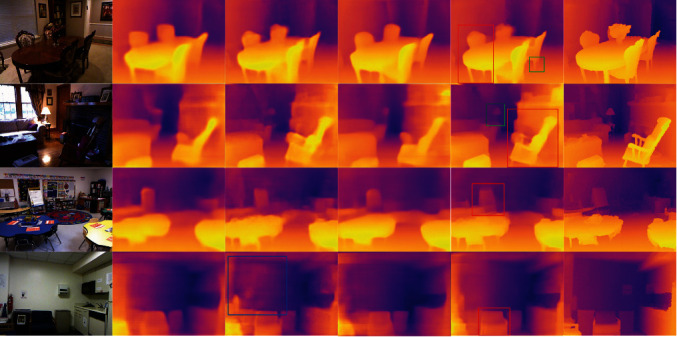
The illustrations of depth estimation comparisons on four images of different frameworks. (a) RGB inputs; (b) PSD; (c) PSD + EESP; (d) PSD + RES; (e) PSD + EESP + RES; (f) ground truth depth maps.

The decoder mainly contains three parts: the EESP connection modules, the PSD modules, and the residual connections. This section verifies the effects of the three modules on the depth estimation performance by ablation experiments. The experimental results are shown in Figure 5. Compared with the predicted depth maps in column (b) of Figure 5, the sharpness of object edges in the predicted depth maps in column (c) of Figure 5 is significantly improved, and the performance of predicting small objects depth values, such as table lamps and chair legs is enhanced, which proves that the EESP connection module can effectively improve the depth prediction performance of the network for object edges and small objects. Comparing the areas of the blue box in column (c) of the last row in Figure 5 with the corresponding areas in columns (b) and (e), it can be seen that the EESP connection modules disrupt the continuity of depth values in the depth prediction maps, while the combination of the EESP modules and the residual connections can effectively improve the continuity of predicted depth values. Compared with the predicted depth maps in columns (b), (c), and (d) in Figure 5, the areas marked in red and green boxes in column (e) reflect that the predicted depth maps obtained by using these modules jointly not only have clearer object contours but also greatly improve the problem of missing small objects.

## 6. Conclusion

A depth estimation encoder-decoder architecture based on spatial pyramid EESP and pixel shuffle is proposed in this paper to address the problems of object distortion and missing small objects existing in monocular depth estimation. The spatial pyramid EESP modules are used to fully utilize the features of different scales. The proposed pixel shuffle decoder upsamples the features extracted from the backbone network and generates the depth prediction map by fusing the features of different scales step by step. Compared with other state-of-the-art methods, the depth map estimated by our method has clearer object contours, less object distortion, and fewer small objects missing. The experimental result demonstrates the role of the EESP connection module and residual connection in feature fusion and verifies the reliability of our method in solving the problem of missing and blurred contours of small objects. However, the depth estimation performance of our method is not desirable in areas of very small depth values and objects refracting light such as mirrors, so the next step will be to try masking and other methods to improve the accuracy of depth estimation.

## Figures and Tables

**Table 1:** Depth estimation comparisons of our methods and other advanced methods.

Method	Errors			Accuracy (%)		
	rel	log10	rms	*δ*_1_	*δ*_2_ (%)	*δ*_3_ (%)
Liu et al. [7]	0.230	0.095	0.824	61.4	88.3	97.1
Xu et al. [25]	0.163	0.069	0.655	70.6	92.5	98.5
Li et al. [26]	0.143	0.063	0.635	78.8	95.8	99.1
Laina et al. [6]	0.127	0.055	0.573	81.1	95.3	98.8
Xu et al. [27]	0.125	0.057	0.593	80.6	95.2	98.1
Wofk et al. [8]	—	—	0.604	77.1	—	—
Cao et al. [28]	0.132	0.057	0.538	83.1	96.2	98.8
Xu et al. [9]	0.149	0.062	0.495	80.5	96.2	—
He et al. [11]	0.145	0.062	0.514	80.5	96.2	99.2
Dong et al. [29]	0.146	—	0.537	79.9	95.1	98.8
Ours	0.141	0.060	0.503	81.3	96.0	99.0

**Table 2:** Comparison of different EESP modules.

Method	Errors		Accuracy (%)		
	rel	rms	*δ*_1_	*δ*_2_ (%)	*δ*_3_ (%)
3-EESP	0.142	0.505	81.2	95.9	98.8
EESP-4	0.144	0.505	80.8	95.8	98.9
EESP-HFF	0.141	0.512	81.3	95.6	98.8
Proposed design	0.141	0.503	81.3	96.0	99.0

**Table 3:** Comparison of different feature fusion methods in PSD.

Method	Errors		Accuracy (%)		
	rel	rms	*δ*_1_	*δ*_2_ (%)	*δ*_3_ (%)
A	0.151	0.522	79.3	95.5	98.9
B	0.147	0.513	80.1	95.8	98.9
C	0.178	0.607	73.4	93.0	98.0
D	0.147	0.515	80.1	95.6	98.9
Proposed	0.141	0.503	81.3	96.0	99.0

## Data Availability

The data used to support the findings of this study are available at https://cs.nyu.edu/∼silberman/datasets/nyu_depth_v2.html.

## References

[1] Zia S., Yuksel B., Yuret D., Yemez Y. Rgb-d object recognition using deep convolutional neural networks.

[2] Lu Y., Zhou J., Wang J. Curve-structure segmentation from depth maps: A cnn-based approach and its application to exploring cultural heritage objects.

[3] Yan T., Wu P., Qian Y., Hu Z., Liu F. (2020). Multiscale fusion and aggregation pcnn for 3d shape recovery. *Information Sciences*.

[4] Bouazzaoui I., Florez S. A. R., El Ouardi A. (2022). Enhancing rgb-d slam performances considering sensor specifications for indoor localization. *IEEE Sensors Journal*.

[5] Eigen D., Puhrsch C., Fergus R. (2014). *Depth Map Prediction from a Single Image Using a Multi-Scale Deep Network*.

[6] Laina I., Rupprecht C., Belagiannis V., Tombari F., Navab N. Deeper depth prediction with fully convolutional residual networks.

[7] Liu F., Shen C., Lin G., Reid I. (2016). Learning depth from single monocular images using deep convolutional neural fields. *IEEE Transactions on Pattern Analysis and Machine Intelligence*.

[8] Wofk D., Ma F., Yang T. J., Karaman S., Sze V. Fastdepth: Fast monocular depth estimation on embedded systems.

[9] Xu X., Chen Z., Yin F. (2021). Monocular depth estimation with multi-scale feature fusion. *IEEE Signal Processing Letters*.

[10] Li K., Fu Z., Wang H., Chen Z., Guo Y. (2021). Adv-depth: Self-supervised monocular depth estimation with an adversarial loss. *IEEE Signal Processing Letters*.

[11] He L., Lu J., Wang G., Song S., Zhou J. (2021). Sosd-net: Joint semantic object segmentation and depth estimation from monocular images. *Neurocomputing*.

[12] He K., Zhang X., Ren S., Sun J. Deep residual learning for image recognition.

[13] Eigen D., Fergus R. Predicting depth, surface normals and semantic labels with a common multi-scale convolutional architecture.

[14] Hu J., Ozay M., Zhang Y., Okatani T. Revisiting single image depth estimation: Toward higher resolution maps with accurate object boundaries.

[15] Odena A., Dumoulin V., Olah C. (2016). Deconvolution and checkerboard artifacts. *Distill*.

[16] Feng X., Li X., Li J. (2021). Multi-scale fractal residual network for image super-resolution. *Applied Intelligence*.

[17] Zhao X., Zhang Y., Zhang T., Zou X. (2019). Channel splitting network for single mr image super-resolution. *IEEE Transactions on Image Processing*.

[18] Sun Y., Chen J., Liu Q., Liu G. (2020). Learning image compressed sensing with sub-pixel convolutional generative adversarial network. *Pattern Recognition*.

[19] Tian Z., He T., Shen C., Yan Y. Decoders matter for semantic segmentation: Data-dependent decoding enables flexible feature aggregation.

[20] Chollet F. Xception: Deep learning with depthwise separable convolutions.

[21] Howard A. G., Zhu M., Chen B. (2017). Mobilenets: Efficient convolutional neural networks for mobile vision applications. http://www.w3.org/2001/XMLSchema:anyURI.

[22] Yu F., Koltun V. (2015). Multi-scale context aggregation by dilated convolutions. http://www.w3.org/2001/XMLSchema:anyURI.

[23] Mehta S., Rastegari M., Caspi A., Shapiro L., Hajishirzi H. Espnet: Efficient spatial pyramid of dilated convolutions for semantic segmentation.

[24] Silberman N., Hoiem D., Kohli P., Fergus R. Indoor segmentation and support inference from rgbd images.

[25] Xu D., Ricci E., Ouyang W., Wang X., Sebe N. Multi-scale continuous crfs as sequential deep networks for monocular depth estimation.

[26] Li J., Klein R., Yao A. A two-streamed network for estimating fine-scaled depth maps from single rgb images.

[27] Xu D., Wang W., Tang H., Liu H., Sebe N., Ricci E. Structured attention guided convolutional neural fields for monocular depth estimation.

[28] Cao Y., Zhao T., Xian K., Shen C., Cao Z., Xu S. (2020). Monocular depth estimation with augmented ordinal depth relationships. *IEEE Transactions on Circuits and Systems for Video Technology*.

[29] Dong X., Garratt M. A., Anavatti S. G., Abbass H. A. (2022). Mobilexnet: An efficient convolutional neural network for monocular depth estimation. *IEEE Transactions on Intelligent Transportation Systems*.

